# Engineering biomimetic coronary artery equivalents for Kawasaki disease modeling and drug screening

**DOI:** 10.1016/j.mtbio.2026.103701

**Published:** 2026-09-22

**Authors:** Xuting Zhang, Changmin Shao, Yanke Wang, Andong Liu, Xing Rong, Chang Jia, Weiting Yu, Hao Zhang, Rongzhou Wu, Jingyi Zhang, Lexiang Zhang, Fangfu Ye, Maoping Chu

**Affiliations:** a Children's Heart Center, Pediatric Interdisciplinary Consortium, The Second Affiliated Hospital and Yuying Children's Hospital of Wenzhou Medical University, Wenzhou, China; b Zhejiang Provincial Clinical Research Center for Pediatric Precision Medicine, Wenzhou, China; c Wenzhou Institute, University of Chinese Academy of Sciences, Wenzhou, China; d Beijing National Laboratory for Condensed Matter Physics, Institute of Physics, Chinese Academy of Sciences, Beijing, China; e Key Laboratory of Structural Malformations in Children of Zhejiang Province, Wenzhou, China; f Pediatric Research Institute, Pediatric Interdisciplinary Consortium, The Second Affiliated Hospital and Yuying Children's Hospital of Wenzhou Medical University, Wenzhou, China

**Keywords:** Kawasaki disease, Microfluidic coaxial bioprinting, Vascular tissue engineering, In vitro disease model, Drug screening

## Abstract

Kawasaki disease (KD) is an acute vasculitis that primarily affects the pediatric population, with coronary artery lesions constituting its most severe clinical sequela. Although investigated for decades, the precise etiology and pathogenesis of this syndrome remain elusive. This critical knowledge gap stems from the absence of robust in vitro platforms capable of accurately recreating the complex pathophysiological dynamics of pediatric coronary arteries. To overcome this challenge, we developed hollow hydrogel fibers using microfluidic coaxial printing, which were then seeded with human coronary artery smooth muscle cells (HCASMCs) and human umbilical vein endothelial cells (HUVECs) to generate a biomimetic coronary artery construct. The physiological responsiveness of the model was validated through stimulation with TNF-α, and a KD-specific pathological model was established by introducing serum from KD patients. Using this biomimetic platform, we evaluated the therapeutic potential of Atorvastatin (ATOR) and the NLRP3 inflammasome inhibitor MCC950, and explored their underlying mechanisms in attenuating vascular inflammation. Overall, this work introduces a structurally relevant in vitro platform that offers new opportunities for studying KD pathogenesis and accelerating the development of targeted therapies.

## Graphical abstract

**Figure:**
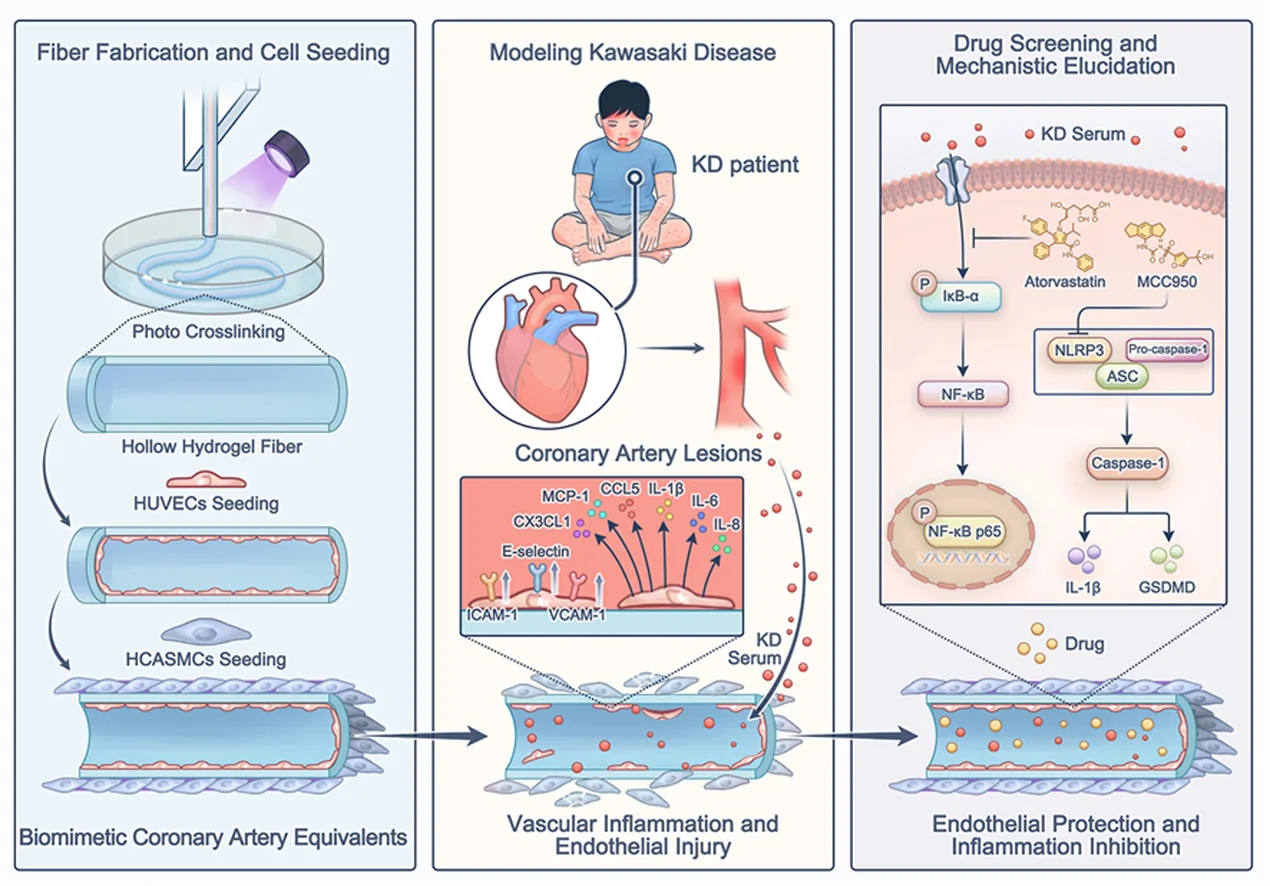

## 1 Introduction

Kawasaki disease (KD), a systemic vasculitis primarily affecting children under five years of age, presents as an acute febrile illness. It is now widely recognized as the leading cause of acquired pediatric cardiovascular disease in developed countries [1,2]. KD's clinical course is often complicated by coronary artery lesions, among which coronary artery aneurysm represents the most severe manifestation, which often results in permanent cardiac impairment [3,4]. Despite its clinical significance, the exact cause of KD remains unknown. The mechanisms contributing to resistance to intravenous immunoglobulin (IVIG) therapy have not been fully elucidated, which presents ongoing difficulties in patient management [5,6]. Therefore, developing reliable experimental models is essential to better understanding KD pathogenesis and identifying novel therapeutic candidates. Although animal models, particularly murine systems have been used to study KD, their relevance to humans is often limited by differences in vascular biology and immune function between species [[7], [8], [9]]. Human cell-based models provide a more genetically and phenotypically representative alternative. However, most current systems are limited to two-dimensional (2D) monocultures or basic static co-cultures [10,11]. Conventional approaches fail to capture the three-dimensional (3D) multilayer architecture and hollow structure characteristic of native coronary arteries. Consequently, it is imperative to engineer 3D in vitro platforms that accurately recreate the human vascular microenvironment in order to facilitate robust pharmacological screening for KD.

Microfluidic coaxial bioprinting has recently gained traction as a promising biofabrication platform capable of generating vessel-like structures that closely mimic native physiology [[12], [13], [14]]. Specifically, it utilizes a coaxial nozzle system to co-deliver multiple bioinks in a laminar flow configuration, enabling the continuous fabrication of self-supporting, hollow hydrogel microtubes via crosslinking [15,16]. The key advantages of this approach lie in its operational simplicity and high throughput, which facilitate the uninterrupted production of tubular constructs under mild, cell-friendly conditions [[17], [18], [19]]. Besides, it supports the use of highly biocompatible materials, thereby preserving an optimal microenvironment for cellular growth and function [[20], [21], [22]]. Recent advancements documented in the literature continually affirm the importance of this fabrication strategy for mimicking human vasculature. For instance, Zhang and his colleagues developed perfusable, hydrogel-based vascular grafts using coaxial printing [23]. Other research teams have used analogous hollow structures to replicate atherosclerotic pathology in controlled, in vitro settings [24]. Collectively, these findings underscore the capacity of microfluidic coaxial bioprinting to engineer complex, 3D hemodynamic microenvironments that faithfully recapitulate native physiological states.

Therefore, we hypothesized that recreating the 3D bilayered architecture of coronary arteries would provide a more physiologically and pathologically accurate platform for modeling KD vasculitis. Unlike conventional in vitro models, our study introduces a novel biomimetic human coronary artery equivalent engineered via microfluidic coaxial bioprinting. By utilizing a highly tunable GelMA/PEGDA hydrogel solution, we constructed perfusable, hollow microtubes with a native-like bilayered cellular architecture. We postulated that exposing this 3D microenvironment to acute KD patient serum would faithfully recapitulate KD-specific endothelial dysfunction and vascular injury. Furthermore, this study distinctively applies this platform for pharmacological screening by evaluating the therapeutic efficacy of Atorvastatin (ATOR) and the targeted NLRP3 inhibitor MCC950, while delineating their distinct regulatory pathways (such as NF-κB and NLRP3 activation). Ultimately, this work provides a reliable, structurally realistic in vitro framework that not only advances our understanding of KD pathogenesis but also establishes a robust preclinical tool for evaluating targeted drugs for KD.

## 2 Results

The overall experimental design and process of this study are shown in Fig. 1. To comprehensively evaluate the engineered vascular model and its application in pathological simulation and drug screening, our detailed results will be systematically presented in the following five sections.

**Fig. 1:**
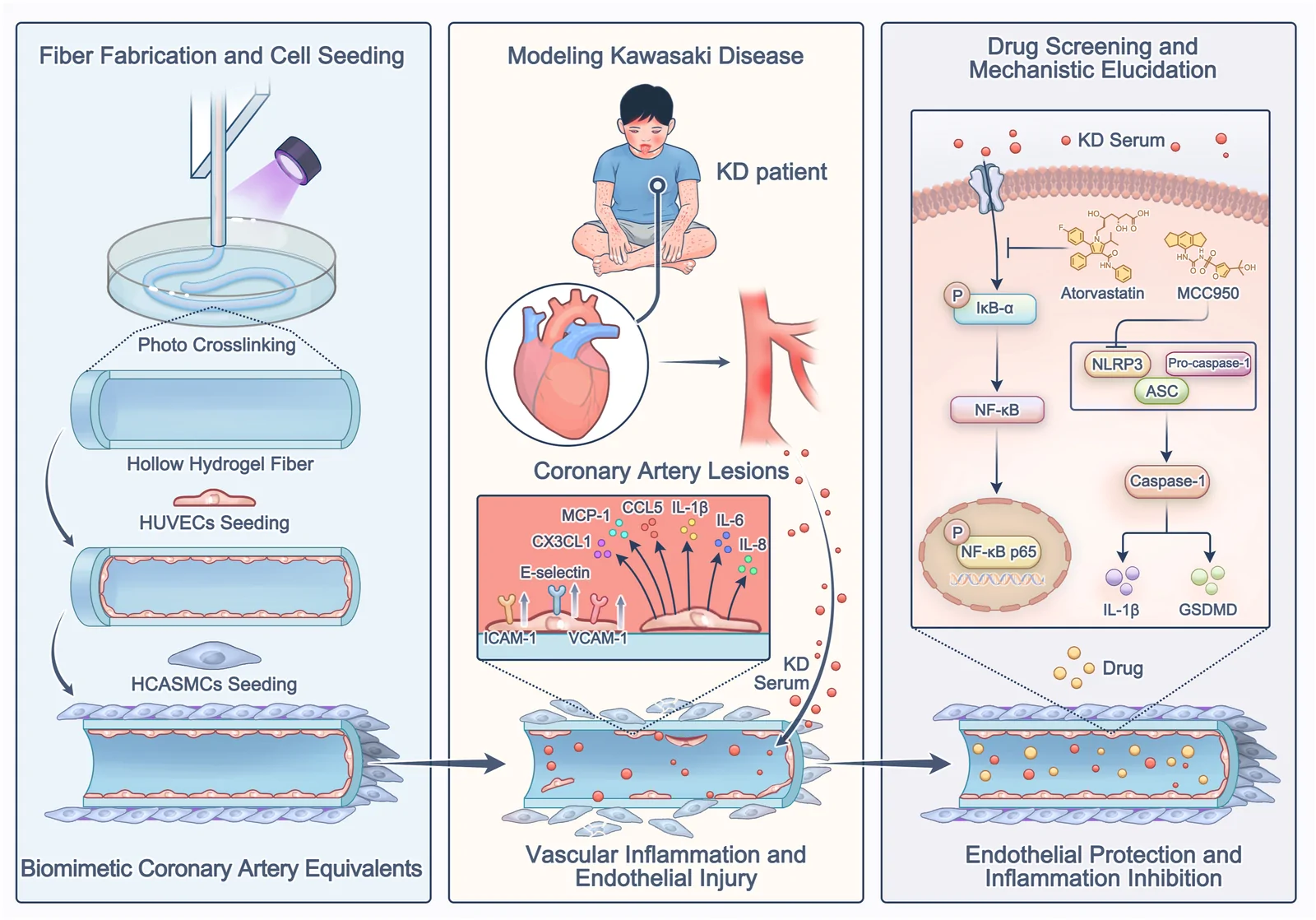
Engineering biomimetic coronary artery equivalents for Kawasaki disease modeling and drug screening.

### 2.1 Fabrication and characterization of hollow hydrogel fibers

We developed a coaxial microfluidic device made of glass capillaries, a glass slide, and injection needles to generate hollow hydrogel fibers. The entire setup was shielded from ambient light during fabrication to prevent premature crosslinking (Fig. S1 and S2). Fig. 2A shows that the outer fluid was a blended bioink containing 10% GelMA and 2.5% PEGDA, while the inner sacrificial phase contained 10% polyvinyl alcohol (PVA). Both solutions were delivered through independent micro-syringe pumps into the coaxial capillary system. Once stable co-flow was achieved at the capillary outlet, the outer hydrogel layer was crosslinked with ultraviolet (UV) exposure, resulting in continuous fiber formation (Fig. 2B). The hollow lumen was then formed by removing the uncrosslinked PVA core (Fig. 2C). Microscopic evaluation using optical and fluorescence imaging confirmed the presence of an open, continuous lumen along the fiber length (Fig. 2D and E). Scanning electron microscopy (SEM) further validated the hollow architecture and revealed a highly porous microstructure within the hydrogel wall (Fig. 2F).

**Fig. 2:**
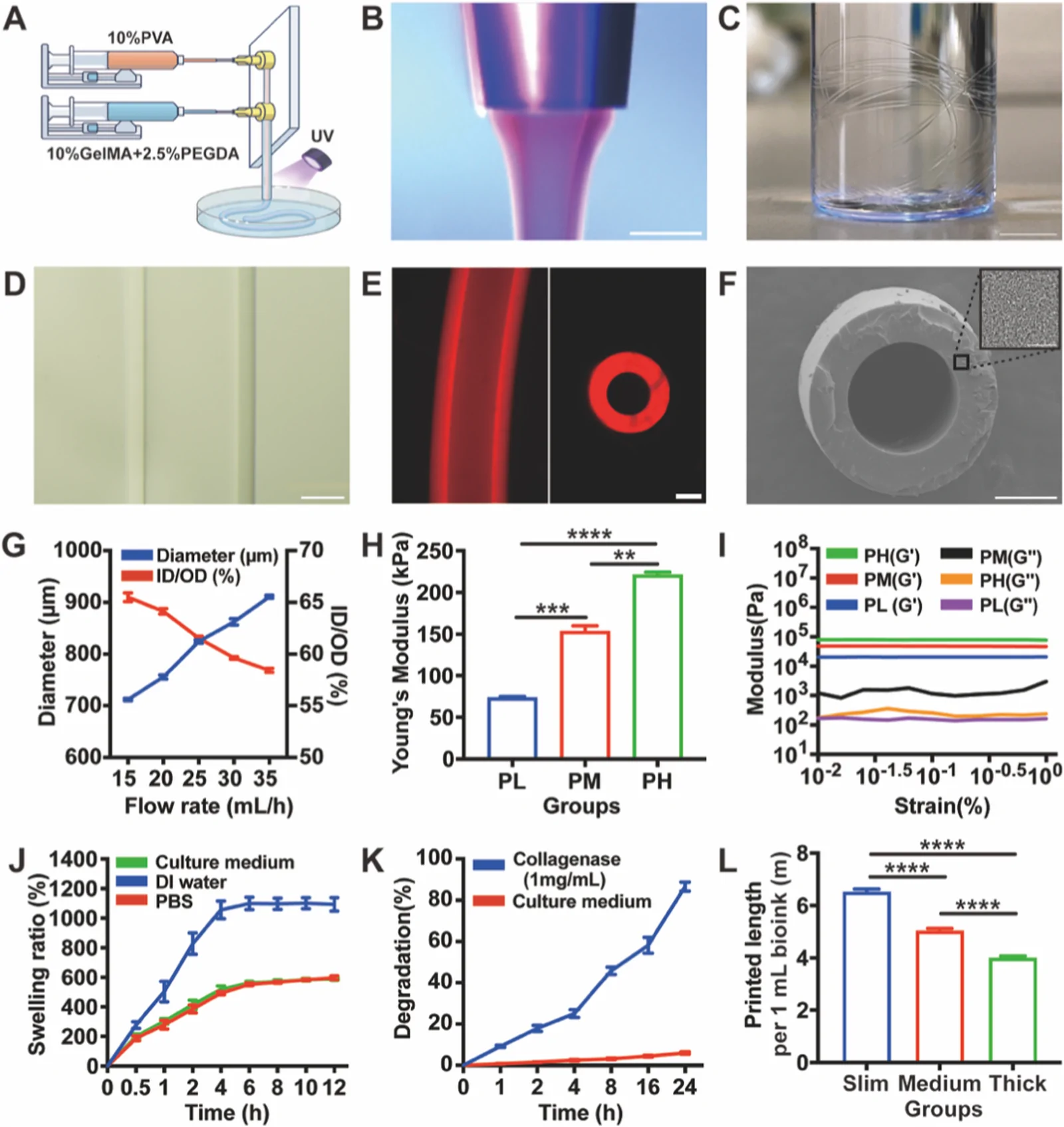
**Fabrication and characterization of hollow hydrogel fibers via microfluidic coaxial bioprinting.** A) Schematic illustration of the microfluidic device used to fabricate the fibers. B) Photopolymerization process of the fibers (scale bar: 500 μm). C) Macroscopic view of the fibers (scale bar: 500 μm). D) Optical microscopy and E) fluorescence microscopy images showing the distinct hollow structure (scale bar: 200 μm). F) SEM images displaying the morphology and porous microstructure of the fibers (scale bar: 200 μm and 2.5 μm [inset]). G) The effect of the external phase flow rate (15-35 mL/h) on fiber dimensions (outer diameter and ID/OD ratio) when the internal phase flow rate is kept at 15 mL/h. H) Young's modulus of hydrogel groups with varying PEGDA concentrations. Groups: PL (2.5% PEGDA), PM (5% PEGDA), and PH (7.5% PEGDA), all of which contain 10% GelMA. I) Rheological analysis showing the storage (G′) and loss (G″) modulus in different groups as a function of strain (0.01–1%). J) Comparative swelling behaviors of the hydrogels in DI water, PBS, and the culture medium. K) Comparative degradation behaviors of the hydrogels in PBS containing collagenase (1 mg/mL) and in culture medium. L) Quantification of the total length of the fibers generated from 1 mL of bioink for the slim (200/400 μm, inner/outer diameters), medium (300/500 μm), and thick (400/600 μm) groups, respectively.

The morphological tunability of the bioprinted fiber was systematically validated by modulating the perfusion kinematics. By fixing the internal fluid velocity at 15 mL/h and elevating the external flow from 15 to 35 mL/h, a quantifiable reduction in the inner/outer diameter (ID/OD) ratio was observed, shifting from 65.50 ± 0.73% to 58.43 ± 0.35%. This hydrodynamic adjustment simultaneously drove an expansion in the macroscopic outer diameter, which broadened from 711.90 ± 3.82 μm to 910.87 ± 5.07 μm (Fig. 2G). In contrast, stabilizing the external flow at 15 mL/h while accelerating the internal flow from 10 to 30 mL/h yielded a concomitant enlargement of both the global construct caliber and the relative luminal cross-section. Specifically, the outer diameter increased from 677.90 ± 4.21 μm to 797.03 ± 5.31 μm, corresponding to a definitive increase in the ID/OD ratio from 63.06 ± 0.88% to 71.84 ± 0.66% (Fig. S3). These findings underscore the fabrication platform's adaptability, enabling the production of hollow fibers with adjustable luminal and wall dimensions suitable for modeling various vascular tissues.

After confirming the structural features, we characterized the mechanical properties of the hollow fibers. To examine the effect of crosslinking density on stiffness, we prepared fibers with three different PEGDA concentrations: 2.5% (PL), 5% (PM), and 7.5% (PH). Tensile testing revealed that Young's modulus increased significantly with rising PEGDA content. Measured values were 73.04 ± 3.25 kPa, 153.00 ± 12.00 kPa, and 220.67 ± 6.43 kPa for the PL, PM, and PH groups, respectively (Fig. 2H). Consistent with the tensile testing results, rheological assessments showed that the storage modulus (G′) and the loss modulus (G″) were highly dependent on the PEGDA concentration (Fig. 2I and S4). For instance, under oscillatory shear conditions (1 Hz, 0.1% strain), the PH group exhibited a storage modulus (G′) of 90.0 ± 1.54 kPa. This value was significantly higher than those recorded for the PM (43.7 ± 0.89 kPa) and PL (17.7 ± 0.52 kPa) groups. These results collectively indicate that the mechanical properties can be precisely controlled by varying the PEGDA content.

Next, we investigated the swelling characteristics and degradation behavior of the hydrogel fibers. As shown in Fig. 2J, submerging the hydrogels in deionized (DI) water, PBS, or the culture media led to accelerated fluid absorption within the first 4 h. A swelling plateau was established within 24 h. Notably, DI water induced the most pronounced volumetric expansion, while the hydration kinetics in PBS and culture media remained similar. Furthermore, degradation assessments confirmed the structural integrity of the matrices, which retained over 95% of their original mass after 24 h in the cell culture media. This indicates robust resistance to hydrolytic breakdown (Fig. 2K). In contrast, exposure to PBS containing collagenase (1 mg/mL) triggered rapid enzymatic breakdown, resulting in complete mass loss within 24 h. This significant difference confirms that fiber degradation is primarily driven by enzymatic activity rather than passive hydrolysis. Lastly, we assessed the fabrication throughput of the system. Using only 1 mL of bioink, we produced continuous hollow fibers of substantial length, depending on the lumen and wall dimensions selected. Specifically, fiber lengths of 6.54 ± 0.17 m, 5.06 ± 0.13 m, and 4.03 ± 0.09 m were achieved for the slim (200/400 μm inner/outer diameters), medium (300/500 μm), and thick (400/600 μm) groups, respectively (Fig. 2L). This high fabrication efficiency underscores the scalability and practical utility of our microfluidic strategy for generating large-scale vascular constructs.

### 2.2 Generation of biomimetic coronary artery equivalents

After establishing the fabrication parameters and characterization of the fibers, we focused on constructing a physiologically relevant coronary artery model through co-culturing HUVECs and HCASMCs. To evaluate whether PEGDA-dependent mechanical differences affected early cell seeding behavior, we compared cell attachment and early growth on hydrogel fibers with different PEGDA concentrations (2.5%, 5%, and 7.5%) over a 48- h culture period (Fig. S5). The results showed that increasing PEGDA concentration influenced initial cell attachment and subsequent cellular coverage. Among the tested formulations, the 10% GelMA + 2.5% PEGDA group provided the most favorable microenvironment for early endothelial attachment and growth. Therefore, this formulation was selected for the fabrication of all subsequent vascular constructs.

To construct the model, the optimized hydrogel fibers were first subjected to UV sterilization. Subsequently, the luminal space was inoculated with a HUVECs suspension (1 × 10^7^ cells/mL) and subsequently maintained in culture for 72 h to facilitate steady cell engraftment and structural expansion. This resulted in the development of a structurally contiguous endothelial monolayer. Next, HCASMCs suspension (1 × 10^8^ cells/mL) were added to the abluminal aspect of the construct. This facilitated the development of a continuous smooth muscle layer. As shown in Fig. 3A, this sequential seeding strategy allowed endothelial cells to progressively cover the luminal surface and smooth muscle cells to successfully colonize the abluminal side. Live/dead staining performed at multiple time points revealed sustained high cell viability (green) and minimal cell death (red), highlighting the engineered microenvironment's cytocompatibility and stability (Fig. 3B).

**Fig. 3:**
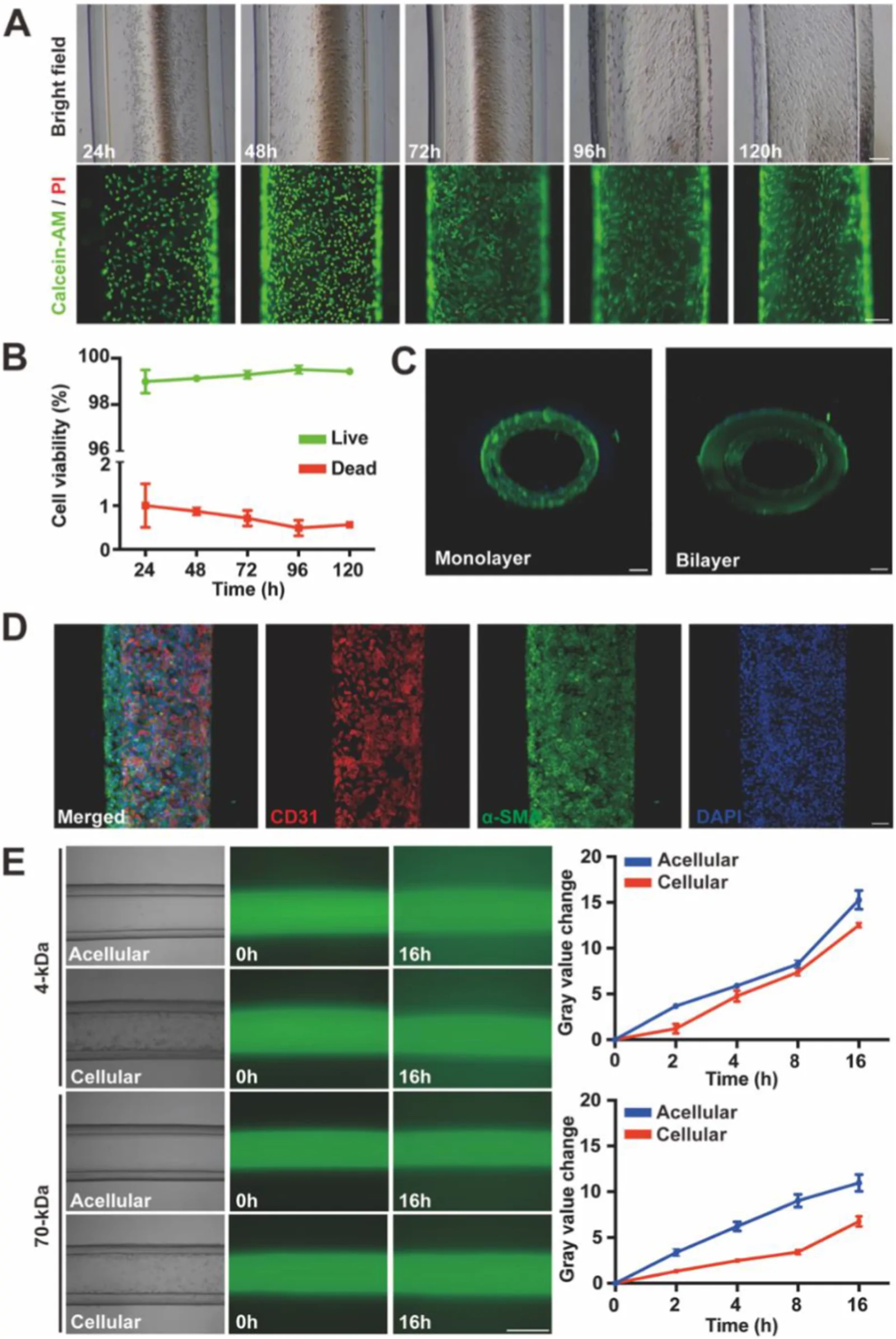
**Establishment and biological characterization of the biomimetic coronary artery equivalents.** A) Representative bright field images showing cell morphology, while fluorescence images depicting cell viability by visually distinguishing viable (green) cells from nonviable (red) cells over a 120 h culture period (24, 48, 72, 96, and 120 h) (scale bar: 100 μm). B) Statistical quantification of cell viability corresponding to each time point. C) Cross-sectional confocal fluorescence images showing the cytoskeletal organization of monolayer and bilayer cellular structures stained for F-actin (FITC-Phalloidin, green) (scale bar: 100 μm). D) Representative fluorescence micrographs demonstrating CD31-positive HUVECs (red) and α-SMA-positive HCASMCs (green), with DAPI for nuclear counterstaining (blue) (scale bar: 100 μm). E) Representative fluorescence images showing the transmural permeation profiles of 4 kDa and 70 kDa FITC-dextran in acellular versus cellular constructs, alongside the corresponding quantitative analysis (scale bar: 500 μm).

To characterize the structural organization of the bilayered construct further, we performed F-actin staining and examined the cytoskeletal architecture using confocal microscopy. 3D reconstruction revealed a clear morphological progression over time. At 72 h, the HUVECs formed a continuous monolayer with uniform circumferential alignment along the lumen. By 120 h, after the addition of the HCASMCs, a distinct bilayer emerged. Both cell types retained their oriented circumferential pattern (Fig. 3C). Immunofluorescence staining was then employed to confirm cellular identity and layer-specific distribution. Fig. 3D shows that CD31, a canonical endothelial marker, was robustly expressed along the luminal surface, while α-smooth muscle actin (α-SMA) was specifically localized to the outer layer. This further validates the successful reconstruction of a vessel-like architecture.

To verify that the construct achieved more than just morphological biomimicry, we subsequently examined the endothelial barrier's functional performance—a fundamental prerequisite for establishing true physiological relevance. We conducted permeability assays by perfusing FITC-labeled dextrans of different molecular weights through the lumen of both cellularized and acellular constructs. In constructs with an intact endothelium, extravascular fluorescence accumulation was significantly reduced compared to acellular controls when challenged with 4 kDa dextran. This reflects the restricted diffusion of small solutes, such as ions and metabolites (Fig. 3E). Similarly, perfusion with 70 kDa dextran demonstrated that the endothelial layer effectively limited macromolecular leakage, mimicking the barrier function of native vessels toward plasma proteins. Together, these findings confirm that the engineered construct replicates the structural hierarchy and essential endothelial barrier properties of the native coronary artery.

### 2.3 Validation of vascular inflammation and endothelial dysfunction

After confirming the structural and functional integrity of the engineered construct, we sought to determine whether it could recapitulate the molecular characteristics of vascular inflammation. To model clinically relevant endothelial dysfunction, we exposed the construct to 10 ng/mL of TNF-α for 6 h. This proinflammatory stimulus led to significant cellular stress, as evidenced by increased lactate dehydrogenase (LDH) release and elevated intracellular total superoxide dismutase (SOD) activity relative to untreated controls (Fig. S8 and S9). Both are indicative of compromised membrane integrity and heightened oxidative burden.

One defining feature of endothelial activation is the surface expression of leukocyte adhesion molecules. Immunofluorescence staining and quantitative analysis showed significant increases in ICAM-1, VCAM-1, and E-selectin levels in the TNF-α group (Fig. 4A and B). These observations were corroborated at the transcriptional level by qRT-PCR analysis. The TNF-α exposure increased the expression of transcripts encoding not only previously identified adhesion molecules, but also proinflammatory cytokines and chemotactic factors, including IL-1β, IL-6, IL-8, CX3CL1, CCL5, and MCP-1 (Fig. 4C).

**Fig. 4:**
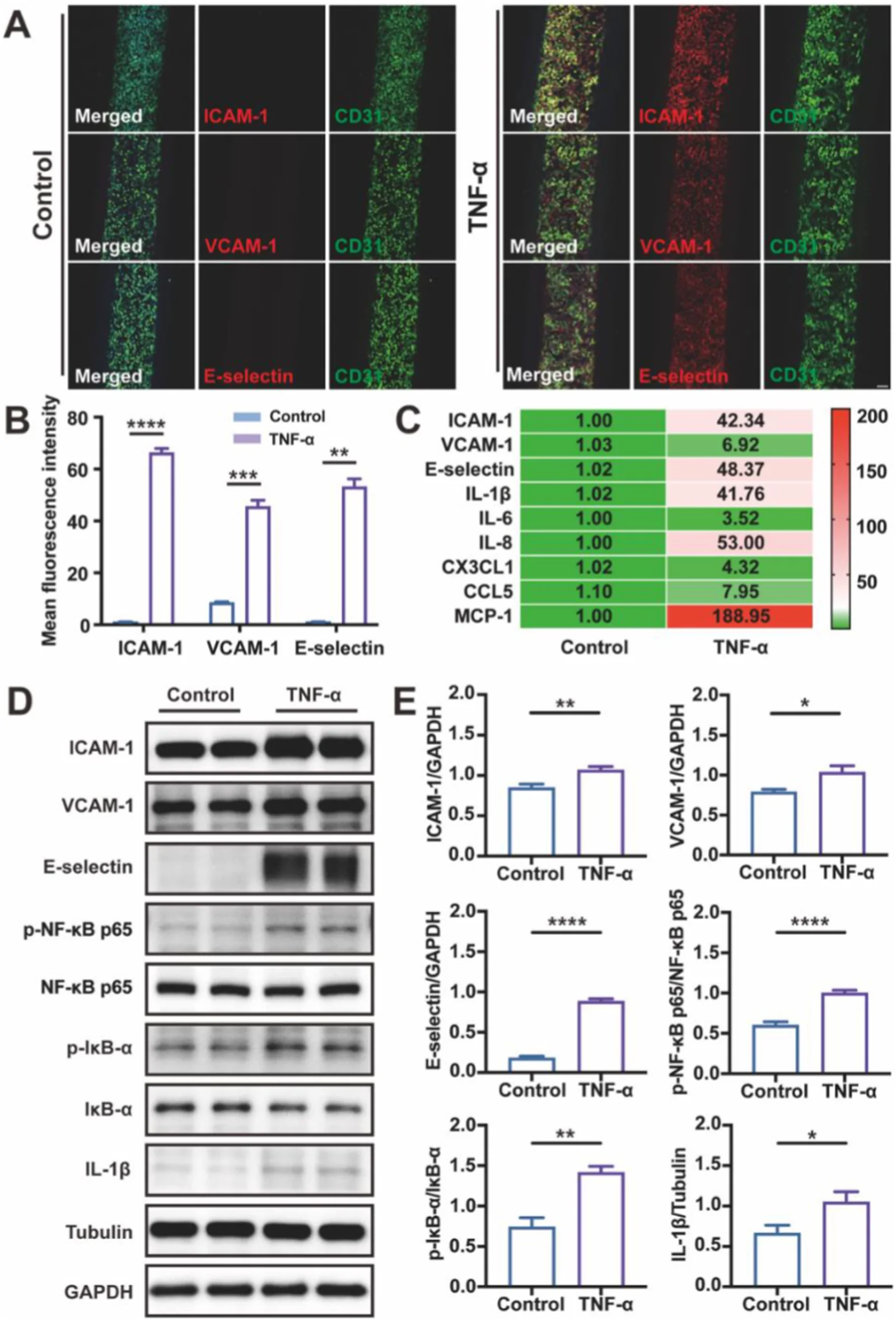
**Validation of TNF-α-induced endothelial dysfunction in the biomimetic coronary artery equivalents.** A) Representative immunofluorescence images showing ICAM-1, VCAM-1, and E-selectin in the control and TNF-α groups (scale bar: 100 μm). B) Quantification of the mean fluorescence intensity from (A). C) Relative mRNA expression levels of indicated adhesion molecules and inflammatory cytokines (ICAM-1, VCAM-1, E-selectin, IL-1β, IL-6, IL-8, CX3CL1, CCL5, and MCP-1). D) Representative WB images and E) corresponding quantitative analysis of ICAM-1, VCAM-1, E-selectin, IL-1β, and NF-κB signaling pathway proteins (p-NF-κB p65, NF-κB p65, p-IκB-α, and IκB-α).

To explore the underlying signaling mechanisms, a Western blot (WB) analysis was performed (Fig. 4D and E). Corroborating our qRT-PCR analysis and immunofluorescent evaluations, exposure to TNF-α drove a marked upregulation of key endothelial adhesion proteins including ICAM-1, VCAM-1, and E-selectin. Parallel to this surface modulation, the inflammatory challenge also engaged the NF-κB signaling axis, a shift clearly delineated by the enhanced phosphorylation of both IκB-α and the p65 subunit. Concomitant upregulation of IL-1β protein provided additional evidence linking NF-κB activation to inflammatory amplification downstream.

Taken together, these results demonstrate that the engineered vascular construct reproduces pathogenic features of endothelial dysfunction under inflammatory stimulation. The model captures the inflammatory cascade consisting of cellular injury, redox imbalance, upregulation of cell adhesion molecules, and activation of the NF-κB signaling pathway. These findings demonstrate this platform as a robust and translatable tool for investigating vascular inflammation and evaluating potential therapeutic interventions.

### 2.4 Modeling Kawasaki disease-associated coronary artery lesions using patient serum

After establishing the responsiveness of our vascular platform to exogenous inflammatory stimuli, we sought to model the complex pathological environment of KD. To this end, we introduced serum derived from pediatric patients who were diagnosed with KD into the culture system and used serum from age-matched healthy children as a control (healthy control, HC). We supplemented the culture medium with the serum at a 1:9 vol ratio (10% v/v), thereby exposing the endothelialized constructs to the systemic inflammatory environment characteristic of KD. A 6-h KD serum stimulation period was selected as an appropriate time window to capture acute KD serum-induced endothelial inflammatory responses, while avoiding extensive cell death and detachment that would compromise downstream molecular analyses.

Relative to the HC group, exposure to KD serum induced significant endothelial injury. This was evidenced by a significant increase in LDH release and elevated intracellular SOD activity, both of which are markers of compromised membrane integrity and oxidative stress (Fig. S8 and S9). Immunofluorescence imaging revealed that various cellular adhesion proteins (e.g., E-selectin alongside ICAM-1 and VCAM-1) were detectable in both groups (Fig. 5A). But quantitative assessments revealed a significant increase in fluorescence intensity for these three proteins in the KD group relative to the HC group (Fig. 5B). The surface adhesion proteins activation was also mirrored at the transcriptional level. qRT-PCR analysis showed that KD serum not only upregulates the mRNA levels of these adhesion molecules, but also drives the robust expression of a diverse array of proinflammatory mediators, encompassing key cytokines and chemokines such as IL-1β, IL-6, IL-8, CX3CL1, CCL5, and MCP-1 (Fig. 5C). Together, these data suggest that KD serum derived from patients recapitulates the multifaceted inflammatory response characteristic of the disease.

**Fig. 5:**
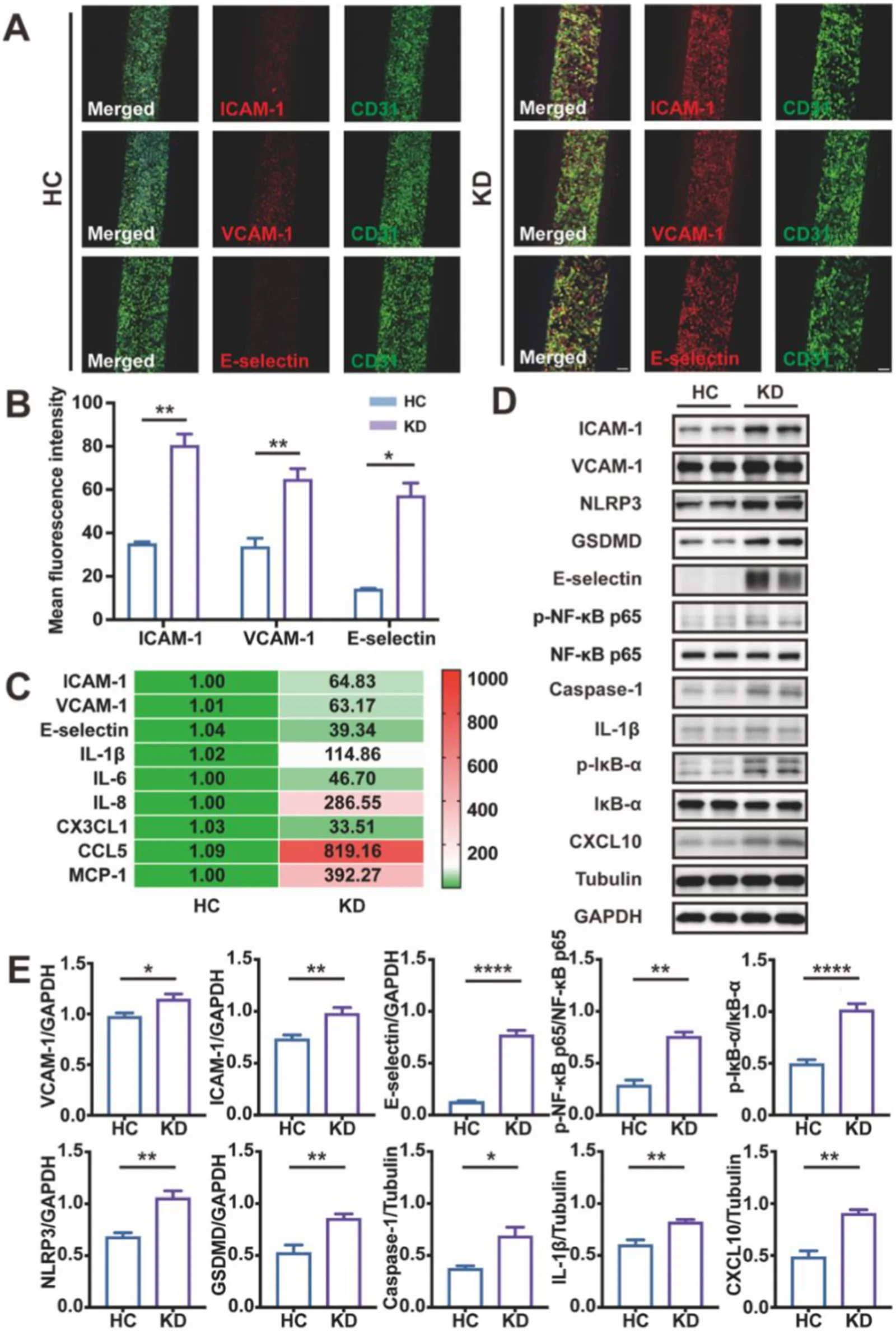
**Modeling of KD-associated coronary artery lesions induced by patient serum.** A) Representative immunofluorescence images showing ICAM-1, VCAM-1, and E-selectin in the HC and KD groups (scale bar: 100 μm). B) Quantification of the mean fluorescence intensity from (A). C) Relative mRNA expression levels of indicated adhesion molecules and inflammatory cytokines (ICAM-1, VCAM-1, E-selectin, IL-1β, IL-6, IL-8, CX3CL1, CCL5, and MCP-1). D) Representative WB images and E) corresponding quantitative analysis of ICAM-1, VCAM-1, E-selectin, NLRP3, GSDMD, p-NF-κB p65, NF-κB p65, Caspase-1, IL-1β, p-IκB-α, IκB-α, and CXCL10.

To investigate the signaling pathways underlying this response, WB analysis was performed. We observed that KD serum induced a marked elevation in the levels of adhesion molecules (ICAM-1, VCAM-1, and E-selectin), alongside increased expression of the pro-inflammatory mediators IL-1β and CXCL10. Consistent with the upregulation of these downstream targets, KD serum stimulation led to robust induction of the NF-κB signaling cascade, which was corroborated by elevated phosphorylation levels of both IκB-α and the p65 subunit (Fig. 5D and E). Given prior reports implicating pyroptosis in KD-associated vascular injury [25], we examined key effectors of this programmed cell death pathway. Analysis revealed a marked upregulation in the protein expression of NLRP3, GSDMD, and caspase-1 within the KD group relative to the HC group (Fig. 5D and E), suggesting that pyroptotic cell death contributes to the observed endothelial damage.

Taken together, these findings demonstrate that, when exposed to patient-derived KD serum, our engineered vascular construct captures the hallmark features of KD vasculitis: inflammatory activation, NF-κB signaling, and pyroptotic cell death. Thus, this disease model provides a physiologically relevant platform for investigating KD mechanisms and screening potential therapeutic interventions.

### 2.5 Evaluation of the therapeutic efficacy of atorvastatin and MCC950

To validate the translational utility of this biomimetic coronary artery platform for pharmacological evaluation, and to elucidate the underlying mechanisms of prospective therapeutics, we first examined the effects of ATOR, a commonly used HMG-CoA reductase inhibitor. Although ATOR is a cornerstone of cardiovascular therapy, its specific clinical efficacy in KD is debated due to conflicting reports. To address this issue, we evaluated its impact within our biomimetic pathological system. We determined the non-cytotoxic working concentration range of ATOR. Based on the calculated IC_50_ profile (Fig. S10), a working concentration of 10 μM was selected for all subsequent biological assays. Pretreatment with ATOR prior to KD serum stimulation significantly attenuated the endothelial inflammatory response. Immunofluorescence imaging revealed a pronounced reduction in ICAM-1 expression following treatment (Fig. 6A and B).

**Fig. 6:**
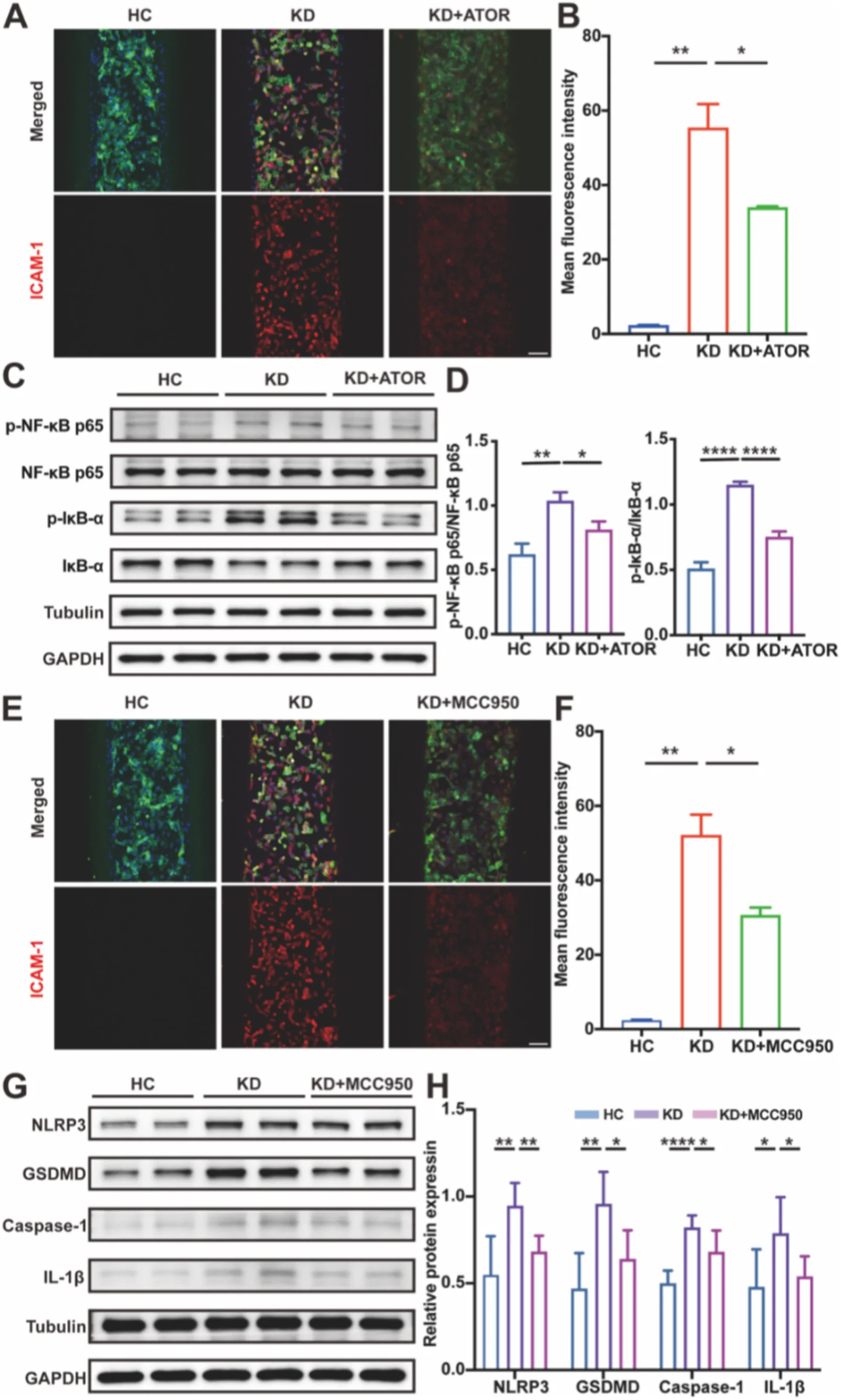
**Evaluation of the therapeutic effects of ATOR and MCC950 in the KD model**. A) Representative immunofluorescence images showing ICAM-1 in the HC, KD, and KD + ATOR groups (scale bar: 100 μm). B) Quantification of the mean fluorescence intensity from (A). C) Representative WB images and D) corresponding quantitative analysis of p-NF-κB p65, NF-κB p65, p-IκB-α, and IκB-α in the HC, KD, and KD + ATOR groups. E) Representative immunofluorescence images showing ICAM-1 in the HC, KD, and KD + MCC950 groups (scale bar: 100 μm). F) Quantification of the mean fluorescence intensity from (E). G) Representative WB images and H) corresponding quantitative analysis of NLRP3, GSDMD, Caspase-1 and IL-1β.

Subsequent qRT-PCR analysis confirmed the observed cellular phenotypes, revealing profound ATOR-induced suppression of the transcriptional expression of key adhesion proteins, including ICAM-1, VCAM-1, and E-selectin. Notably, compared with the untreated KD group, ATOR intervention markedly downregulated the transcript levels of a broad spectrum of pro-inflammatory cytokines and chemokines (IL-1β, IL-6, IL-8, CX3CL1, CCL5, and MCP-1) (Fig. S11). Immunoblotting assays revealed that ATOR potently inhibited the NF-κB cascade, as evidenced by a significant decrease in the phosphorylation status of IκB-α and NF-κB p65 (Fig. 6C and D). Taken together, these data suggest that ATOR mediates cellular protection against KD serum-induced endothelial damage primarily by dampening NF-κB signaling.

Based on the growing body of evidence linking endothelial pyroptosis to KD pathogenesis, we evaluated the therapeutic efficacy of MCC950, a selective NLRP3 inflammasome inhibitor. Similarly, we also determined the non-toxic working concentration of MCC950, and all subsequent interventions were conducted at a concentration of 10 μM (Fig. S10). Similar to the protective effects observed with ATOR, immunofluorescence imaging revealed that MCC950 treatment significantly reduced ICAM-1 protein expression (Fig. 6E and F). Consistently, qRT-PCR analysis revealed substantial downregulation of endothelial adhesion molecule and inflammatory mediator mRNA abundance compared to the KD group (Fig. S12). WB analysis revealed that MCC950 robustly inhibited the classical pyroptotic axis. This was demonstrated by the significant downregulation of key effector proteins, including NLRP3, GSDMD, and caspase-1 (Fig. 6G and H). Taken together, these findings suggest that blocking the NLRP3 inflammasome and its downstream execution phase with MCC950 provides significant protection to the endothelium during KD serum exposure.

## 3 Discussion

In this research, we successfully engineered a physiologically relevant, 3D vascular model using microfluidic coaxial bioprinting to investigate the pathogenesis of KD. Unlike traditional static cultures, our hollow fiber constructs recapitulated the native multilayered architecture and selective barrier function of human coronary arteries. By introducing patient-derived serum, we successfully simulated the specific pathological microenvironment of KD, revealing that endothelial dysfunction in this context is characterized by robust NF-κB pathway activation and the concurrent induction of pyroptosis. Beyond mere structural replication, the translational potential of this microengineered platform was further underscored by its capacity to support pharmacological screening. The successful validation of ATOR and the NLRP3 inhibitor MCC950 highlights the system's exceptional capacity to dissect concurrent pathological pathways and streamline the discovery of novel therapeutics.

Establishing robust in vitro models that faithfully replicate human vascular pathophysiology remains a fundamental challenge in translational research [26]. The microfluidic coaxial bioprinting strategy presented here addresses this need by enabling the fabrication of perfusable, freestanding tubular constructs with precisely tunable dimensions—a significant advancement over conventional 2D monocultures [27]. While 2D systems have historically provided valuable insights into endothelial biology, their planar geometry fails to recapitulate the curved architecture and multicellular interactions inherent to native vessels, often resulting in aberrant cellular responses [28]. Compared to animal models which are constrained by significant interspecies differences and ethical concerns, this engineered human cell system closely resembles native human physiology. Moreover, the establishment of a functional endothelium exhibiting tight junction integrity enables accurate modeling of hemodynamic barrier properties essential for studies of vascular permeability and transendothelial transport.

To construct the biomimetic vascular equivalent, a composite bioink comprising GelMA and PEGDA was utilized in this study. GelMA was selected as the primary component due to its widespread application in tissue engineering and exceptional biocompatibility, providing essential native-like extracellular matrix (ECM) cues that promote cell adhesion and spreading [29,30]. However, pure GelMA hydrogels often lack the structural fidelity required to maintain self-supporting, perfusable micro-tubes. PEGDA was therefore incorporated to provide rapid photo-crosslinking capability and tunable mechanical strength [31]. However, PEGDA has been proven to have the property of resisting cell adhesion [32,33]. Therefore, we optimized the concentration of PEGDA, ensuring both the mechanical strength of the biomimetic vascular equivalent and facilitating cell adhesion and proliferation. Consequently, the formulation containing 10% GelMA and 2.5% PEGDA was ultimately selected, as it provided the optimal balance between maintaining structural integrity and fostering a highly biocompatible microenvironment. Recently, the development of “smart” bioinks, including tissue-specific decellularized extracellular matrix (dECM) bioinks, reversible bioinks, and microgel-based biphasic bioinks, has provided new opportunities to endow engineered vascular tissues with more complex, dynamically responsive, and physiological properties [34,35]. Moving forward, integrating these advanced, tissue-specific smart materials with our microfluidic coaxial bioprinting platform holds great promise.

Building upon this structural foundation, a biological innovation of this work lies in the use of patient-derived serum to induce a disease-specific endothelial phenotype. It should be explicitly noted that while TNF-α was utilized in initial assays solely as a general positive control to validate the functional responsiveness of our engineered construct, it does not represent the full spectrum of KD pathology. In fact, KD is pathophysiologically characterized by a systemic cytokine storm—a complex inflammatory milieu that cannot be adequately reproduced by isolated factor stimulation [36]. By introducing the patient-derived serum, we successfully captured the authentic signaling landscape operative in the diseased vasculature. Within this pathologically relevant microenvironment, we identified the NF-κB cascade as a critical mediator of endothelial injury. This observation aligns with the established consensus that NF-κB dysregulation constitutes a central pathogenic driver across various systemic vasculitides, with prior studies specifically implicating this pathway in KD [37]. Our findings substantiate this paradigm within a controlled 3D setting: treatment with KD serum markedly induced the phosphorylation of both IκB-α and the NF-κB p65 subunit, which drives a pronounced transcriptional amplification of downstream gene targets, notably encompassing chemokines and key adhesion proteins such as ICAM-1 and VCAM-1. The faithful reproduction of this canonical signaling event underscores the biological fidelity of our microengineered platform and its capacity to mirror core inflammatory mechanisms underlying KD vasculitis.

Extending beyond classical inflammatory pathways, our investigation focused on delineating the contribution of pyroptosis to KD-associated endothelial injury. While apoptosis has traditionally received considerable attention in vasculitis research, we observed markedly elevated levels of the pyroptosis-executing protein GSDMD, accompanied by NLRP3 inflammasome activation and Caspase-1 cleavage, in constructs exposed to KD serum. These findings corroborate and extend the previous work implicating pyroptosis in KD pathogenesis [38,39]. Exposure to serum from patients with KD concurrently triggers both NF-κB signaling and pyroptotic cascades, pointing toward a sophisticated crosstalk linking these two mechanisms. Instead of a strictly sequential cascade, NF-κB signaling likely works together with the NLRP3 inflammasome to prime it, thereby exacerbating a pathogenic loop of sustained inflammation and cellular mortality [40,41].

The translational relevance of our model was further validated through pharmacological intervention studies. Treatment with ATOR significantly attenuated endothelial activation and reduced NF-κB phosphorylation. Although statins are clinically indicated primarily for cholesterol reduction, our findings provide additional support for their well-documented pleiotropic effects, particularly their potent anti-inflammatory properties independent of lipid-lowering activity [42]. These observations offer in vitro evidence supporting the potential repurposing of statins as adjunctive therapy for KD patients, especially those exhibiting refractory inflammation despite standard treatment [43,44]. Furthermore, the pronounced protective effect conferred by MCC950, a selective NLRP3 inhibitor, highlights the therapeutic promise of targeting the pyroptotic machinery [45]. Given that IVIG resistance remains a significant clinical challenge, interventions directed at the specific molecular effectors of inflammatory cell death may provide alternative therapeutic avenues for patients who do not respond to conventional immunomodulatory approaches.

Although the current results are encouraging, it is important to acknowledge certain methodological limitations to identify critical areas for future research. Foremost among these is the static culture conditions under which the vascular constructs were maintained. Although the microfluidic coaxial bioprinting technique successfully generated perfusable hollow geometries, endothelial cells were not subjected to continuous fluid flow or physiological shear stress during the experimental period. Hemodynamic forces, particularly wall shear stress, represent critical biomechanical cues that modulate endothelial phenotype, alignment, and inflammatory responsiveness. Future iterations of this model will therefore incorporate dynamic perfusion systems to introduce controlled fluid flow, enabling more accurate recapitulation of the complex hemodynamic environment characteristic of coronary circulation. Such advances will facilitate investigation into how aberrant hemodynamics may synergize with soluble inflammatory mediators to drive KD pathogenesis, further enhancing the physiological relevance of the platform. Another limitation is that HUVECs were utilized as the endothelial layer rather than human coronary artery endothelial cells (HCAECs). While HCAECs are physiologically more specific to coronary arteries, HUVECs were selected due to their robust proliferative capacity, which is essential for forming an intact, confluent endothelial barrier within a 3D bioprinted hydrogel scaffold—a process where primary HCAECs often exhibit limited expansion and survival [46,47]. Furthermore, HUVECs represent the most extensively validated in vitro model for KD research, effectively recapitulating key endothelial inflammatory responses and dysfunction when challenged with KD patient serum [25,48]. Future iterations of this model will aim to optimize the 3D microenvironment to better support the co-culture of primary HCAECs and HCASMCs for enhanced physiological mimicry.

Nevertheless, even in its current static configuration, the engineered vascular construct represents a structurally biomimetic and functionally robust tool for elucidating molecular mechanisms and evaluating potential therapeutic candidates.

## 4 Conclusion

In summary, this research demonstrates the successful development of the 3D biomimetic vascular model using microfluidic coaxial bioprinting technology. After sequential cell seeding, we fabricated hollow hydrogel fibers which can mimic the multilayered structure and barrier function of native coronary arteries. This engineered platform overcomes the geometric and functional limitations of conventional 2D monolayer cultures, providing a more physiologically relevant system for vascular research. Critically, we validated this model's fidelity in reproducing the specific pathophysiology of KD. Administration of patient-derived serum induced a clinically relevant in vitro model of endothelial dysfunction, marked by simultaneous pyroptosis and robust NF-κB cascade activation. These observations corroborate the critical involvement of such pathways in the progression of KD vasculitis. Moreover, the efficacy of this platform as a preclinical drug evaluation tool was firmly validated through subsequent pharmacological testing. We verified the protective effects of atorvastatin and the selective NLRP3 inhibitor MCC950, providing in vitro evidence that supports their potential as therapeutic interventions to mitigate endothelial damage. Together, these findings establish a versatile, physiologically relevant experimental system that advances our understanding of KD pathogenesis and serves as a powerful tool for discovering and evaluating novel therapeutic strategies.

## 5 Materials and methods

### 5.1 Construction of microfluidic devices

The microfluidic device consisted of a glass slide, two capillary tubes, and two injection needles assembled into a coaxial nozzle system. A smaller inner capillary was inserted into the lumen of a larger outer capillary. Their tips were aligned concentrically and secured in place with epoxy adhesive. To enable fluid delivery, the proximal ends of both capillaries were connected to the injection needles, which served as inlets. The inlets were also sealed with epoxy to ensure leak-proof operation.

### 5.2 Preparation of bioinks

The photo-crosslinkable precursor was formulated by dissolving GelMA (10% w/v, Shuhe Biotechnology), PEGDA (2.5%, 5%, or 7.5% w/v; Sigma-Aldrich), and LAP photoinitiator (0.25% w/v, Aladdin) in PBS. The sacrificial core stream was generated using polyvinyl alcohol (PVA; Aladdin), which was dissolved in PBS to create a 10% (w/v) solution.

### 5.3 Microfluidic fiber formation

Prior to the experiments, the microfluidic device was shielded from ambient light to prevent unintended photo-crosslinking. To eliminate air bubbles from the system, DI water was initially perfused through the polyethylene (PE) tubing connected to both inlets. Subsequently, the hydrogel precursor solution (shell flow) and the PVA solution (core flow) were infused into the outer and inner capillary channels, respectively, using syringe pumps (LSP01-3A, Longer Precision Pump Co., Ltd). When a stable coaxial laminar flow was established at the capillary outlet, the stream was irradiated with UV light (405 nm) to initiate in situ crosslinking. The resulting hydrogel fibers were collected in PBS and rinsed extensively to remove any residual reagents.

### 5.4 Fluorescent nanoparticle incorporation and imaging

Red fluorescent nanoparticles (excitation/emission: 580/605 nm; Sigma-Aldrich) were dispersed into the hydrogel precursor (1% v/v) via vortexing and sonication prior to standard fiber fabrication. To assess nanoparticle distribution, transverse fiber sections were imaged using an inverted fluorescence microscope (Axio Observer, Carl Zeiss).

### 5.5 Scanning electron microscopy

For SEM analysis, fabricated fibers were dehydrated in a graded ethanol series (70–100% v/v, 30 min/step) and critical-point dried (CPD300, Leica). Samples were subsequently mounted, sputter-coated with gold-palladium (EM ACE600, Leica), and imaged at 5 kV using a field-emission SEM (SU8010, Hitachi) to evaluate surface and internal morphologies.

### 5.6 Tensile testing

Tensile properties were evaluated using a universal testing machine (Instron 5944, Instron). Hydrogels were cast into rectangular strips (40 × 4 × 0.5 mm) to facilitate standardized measurement. The Young's modulus was derived from the initial linear elastic portion (0%–10% strain) of the stress–strain profile, which was generated by subjecting the materials to a constant elongation rate of 1 mm/min. At least three replicates were tested per group.

### 5.7 Rheological characterization

We evaluated the dynamic mechanical properties of the formulations at 25 °C using a Discovery HR-3 rotational rheometer (TA Instruments) with a 25-mm parallel-plate geometry and a predefined measuring gap of 200 μm. To delineate the linear viscoelastic regime (LVR) and capture the evolution of the storage (G′) and loss (G″) moduli, an initial amplitude sweep was applied from 0.01% to 1% strain at a constant frequency of 1 Hz. After establishing the LVR, the frequency-dependent viscoelastic profile was probed by performing a frequency sweep from 0.1 to 10 Hz at a fixed strain of 0.1%. To ensure statistical robustness, a minimum of three specimens were analyzed for each experimental group.

### 5.8 Mass swelling test

The swelling behavior of disc-shaped hydrogels (8 mm diameter) was evaluated in DI water, PBS, and cell culture medium. Lyophilized samples (initial weight W_0_​) were immersed in each medium at room temperature. At predetermined intervals (0.5–12 h), the mass of the swollen sample (W_t_) was recorded after the surface moisture was removed with Kimwipes. The following formula was used to calculate the swelling ratio (SR):

$$\text{SR}=\frac{W_{t}-W_{0}}{W_{0}}\times 100\%$$

### 5.9 Degradation test

The degradation of hydrogels was evaluated in a cell culture medium and in PBS containing 1 mg/mL of collagenase II (ST2303, Beyotime). Lyophilized, disc-shaped samples (8 mm in diameter, initial weight W_0_) were equilibrated in PBS before being transferred to the degradation media. At predetermined time intervals (1-24 h), the samples were retrieved, rinsed with deionized (DI) water, then dried and weighed (W_t_). The following formula was used to calculate the degradation ratio (DR):

$$\text{DR}=\frac{W_{0}-W_{t}}{W_{0}}\times 100\%$$

### 5.10 Assessment of fiber permeability

To assess permeability, the fabricated fibers were bilaterally anchored within a custom polydimethylsiloxane (PDMS) chamber. Fluorescent tracers—specifically, 4 kDa and 70 kDa FITC-dextran formulated at 20 μg/mL—were continuously infused into the luminal inlet via a micro-pump, maintaining a steady flow velocity of 5 mL/h. The ensuing diffusion kinetics were recorded using an inverted fluorescence microscope, with micrographs captured at predetermined intervals (0, 2, 4, 8, and 16 h). Finally, quantitative assessment of the barrier integrity was achieved by analyzing the fluorescent signal intensities across the captured frames employing ImageJ software.

### 5.11 Cell culture

HUVECs (ZQXZ) and HCASMCs (Pricella) were maintained in their respective complete media (ECM, ScienCell; SMCM, Pricella) at 37 °C with 5% CO_2_. Culture media were replenished every other day. Once the cell monolayers attained approximately 80–90% confluency, routine subculturing was performed. Enzymatic detachment was facilitated by a 0.25% trypsin-EDTA solution (Gibco), strictly adhering to the vendor's recommended protocol.

### 5.12 Cell viability assay

Cytotoxicity was evaluated using a Cell Counting Kit-8 (CCK-8; C0037, Beyotime) assay. Cells were seeded in 96-well plates at 5 × 10^3^ cells/well. After attachment, the medium was replaced with sample extracts, which were prepared by incubating UV-sterilized fibers in culture medium for 24 h at 37 °C. After 24, 48, and 72 h of incubation, cells were treated with 10% CCK-8 solution for 30 min, and absorbance at 450 nm was measured using a microplate reader (BioTek). For viability visualization, samples were stained with Calcein-AM and propidium iodide (PI; C1371S, Beyotime) for 30 min at 37 °C in the dark. Fluorescence images were captured using an inverted fluorescence microscope, and the live/dead cell ratio was quantified via ImageJ software.

### 5.13 Cell seeding

The fibers were cut into 3-4 cm segments and UV-sterilized for 30 min. To establish the endothelial lining, HUVECs (1 × 10^7^ cells/mL) were carefully injected into the luminal space of the fibers using a micropipette. The cell-laden constructs were then maintained under static culture conditions to allow for natural cell attachment and uniform growth. When the cells achieved 70-80% confluence (approximately 72 h), HCASMCs (1 × 10^8^ cells/mL) were added dropwise directly onto the outer surface of the fibers using a micropipette. The resulting bilayered constructs were further cultured to allow the formation of a continuous smooth muscle cell layer.

### 5.14 Sample collection and ethics

Venous blood was collected from all subjects via standard phlebotomy. Importantly, for the KD group, all blood samples were specifically collected during the acute phase of the disease, strictly prior to the administration of IVIG therapy. To obtain serum, the peripheral blood samples were centrifuged at 3000 RPM for 15 min, after which they were preserved at −80 °C. This study received approval from the Ethics Committee of the Second Affiliated Hospital of Wenzhou Medical University (No. 2025–K–213–01) and complied with the Declaration of Helsinki. All participants or their legal representatives provided written informed consent. The detailed baseline demographic and clinical characteristics of the enrolled KD patients and healthy controls are summarized in Table S2.

### 5.15 Immunofluorescence and F-actin staining

Preparation of the fiber for immunofluorescence involved initial fixation in 4% paraformaldehyde (PFA), followed by cellular permeabilization utilizing 0.5% Triton X-100. Then, 5% bovine serum albumin (BSA) was applied for non-specific blocking. Filamentous actin (F-actin) architecture was delineated by exposing the samples to FITC-phalloidin (1:200) for 30 min. To profile target proteins, the specimens underwent an overnight incubation with designated primary antibodies (1:400); subsequent detection was achieved using Alexa Fluor-conjugated secondary antibodies (1:400). Following a 30 min nuclear counterstain with DAPI, the specimens were imaged utilizing the confocal microscope (A1R, Nikon). 3D morphological reconstructions were generated via Z-stack capture, and quantitative assessments of the fluorescence signals were conducted utilizing ImageJ software.

### 5.16 Inflammatory stimulation of biomimetic coronary artery equivalents

In the TNF-α treatment group, the biomimetic coronary artery equivalents were cultured for 6 h in a medium supplemented with 10 ng/mL of TNF-α (Novoprotein, Suzhou, China). For the serum-induced models, the biomimetic coronary artery equivalents were incubated for 6 h in a culture medium mixed with either KD patient serum or healthy control (HC) serum at a 1:9 vol ratio (yielding a final serum concentration of 10%).

### 5.17 Drug treatment

ATOR and MCC950 were purchased from MedChemExpress (MCE, USA) and dissolved in dimethyl sulfoxide (DMSO) to prepare 10 mM stock solutions. To determine non-cytotoxic working concentrations and rule out high-dose cytotoxicity, dose-response cell viability assays were first performed. HUVECs were treated with serial concentrations of ATOR or MCC950 for 12 h, and cell viability was assessed using a CCK-8 assay. Dose-toxicity response curves were then generated, and IC_50_ values were calculated accordingly. The engineered 3D vascular constructs were pretreated with the diluted drugs for 6 h. Following this pretreatment phase, the constructs were co-incubated with the medium containing both the respective drugs and the acute-phase KD patient serum.

### 5.18 Assessment of LDH release

To assess cytotoxicity, culture supernatants were collected and centrifuged to clear cellular debris. We quantified extracellular LDH levels relying on a commercial Cytotoxicity Assay Kit (C0016, Beyotime) as directed by the manufacturer. Absorbance readings were obtained at 490 nm via a microplate reader.

### 5.19 Intracellular SOD activity measurement

A WST-8 assay kit (S0101S, Beyotime) was utilized to monitor total SOD function. In brief, treated cells were collected and subjected to lysis. The resulting clarified supernatants were then transferred to a microplate spectrophotometer to record optical density at 450 nm. Enzymatic activity was ultimately derived using the computational methods provided in the assay kit's guidelines.

### 5.20 RNA isolation and quantitative real-time PCR (qRT-PCR)

RNA was isolated using an RNA isolation kit (RC102-2, Vazyme) and its purity and concentration were confirmed via NanoDrop (Thermo Fisher Scientific). cDNA was synthesized using the PrimeScript RT kit (RR360A, Takara Bio). Quantitative PCR was performed on a Bio-Rad CFX96 system using TB Green Premix Ex Taq (RR420A, Takara Bio). Cycling conditions consisted of initial denaturation at 95 °C (30s), followed by 40 cycles of 95 °C (5s) and 60 °C (30s). Melting curve analysis verified amplicon specificity, and mRNA levels were determined using the 2^−ΔΔCt^ method, normalized to GAPDH.

### 5.21 Western blotting

To prepare whole-cell lysates, samples were homogenized in RIPA buffer (P0013B, Beyotime) with protease/phosphatase inhibitors (P1045, Beyotime). Protein concentrations were determined using a BCA assay (P0010, Beyotime). Equal amounts of protein (10 μg) were resolved by SDS-PAGE and transferred to PVDF membranes (Millipore). After blocking in 5% non-fat dry milk, membranes were incubated with primary antibodies (1:1000) overnight at 4 °C, followed by corresponding HRP-conjugated secondary antibodies (1:1000). Protein bands were detected with ECL Prime substrate (P0018S, Beyotime) and analyzed densitometrically via ImageJ, using GAPDH or tubulin for normalization.

### 5.22 Statistical analysis

All numerical data are presented as the mean ± standard deviation (SD). We performed the statistical evaluations utilizing GraphPad Prism version 10 (GraphPad Software). Prior to the pairwise comparison of experimental groups, datasets were assessed for a Gaussian distribution. For normally distributed variables, statistical differences were determined via an unpaired Student's t-test (assuming equal variances) or Welch's *t*-test (for unequal variances). Conversely, non-normally distributed data were analyzed employing the non-parametric Mann-Whitney *U* test. Statistical significance was defined at an alpha level of p < 0.05, with the specific levels of significance denoted as follows: *p < 0.05, **p < 0.01, ***p < 0.001, and ****p < 0.0001.

## CRediT authorship contribution statement

**Xuting Zhang:** Writing – review & editing, Writing – original draft, Resources, Project administration, Investigation, Data curation. **Changmin Shao:** Writing – review & editing, Validation, Supervision, Conceptualization. **Yanke Wang:** Methodology, Investigation, Formal analysis. **Andong Liu:** Software, Project administration, Methodology. **Xing Rong:** Visualization, Validation, Supervision. **Chang Jia:** Visualization, Formal analysis, Conceptualization. **Weiting Yu:** Visualization, Validation, Methodology. **Hao Zhang:** Methodology, Data curation. **Rongzhou Wu:** Validation, Supervision. **Jingyi Zhang:** Data curation. **Lexiang Zhang:** Formal analysis, Conceptualization. **Fangfu Ye:** Resources, Funding acquisition, Conceptualization. **Maoping Chu:** Resources, Funding acquisition, Formal analysis, Conceptualization.

## Declaration of competing interest

The authors declare that they have no known competing financial interests or personal relationships that could have appeared to influence the work reported in this paper.

## Data Availability

Data will be made available on request.
