# Supplementary material for: Engineering biomimetic coronary artery equivalents for Kawasaki disease modeling and drug screening
Source: Mater Today Bio. 2026 Sep 22;41:103701. doi: 10.1016/j.mtbio.2026.103701 (PMC13639735; doi:10.1016/j.mtbio.2026.103701)
Supplement: Supplementary file 1 [file mmc1.docx]

**Supporting Information**

**Engineering Biomimetic Coronary Artery Equivalents for Kawasaki Disease Modeling and Drug Screening**

Xuting Zhang,^1,2,#^ Changmin Shao,^3,#,^* Yanke Wang,^1,2^ Andong Liu,^4^ Xing Rong,^1,2,5^ Chang Jia,^6^ Weiting Yu,^6^ Hao Zhang,^6^ Rongzhou Wu,^1,2,5^ Jingyi Zhang,^1,2^ Lexiang Zhang,^2,3^ Fangfu Ye,^3,4,^* Maoping Chu^1,2,5,^*

1 Children's Heart Center, Pediatric Interdisciplinary Consortium, The Second Affiliated Hospital and Yuying Children's Hospital of Wenzhou Medical University, Wenzhou, China.

2 Zhejiang Provincial Clinical Research Center for Pediatric Precision Medicine, Wenzhou, China.

3 Wenzhou Institute, University of Chinese Academy of Sciences, Wenzhou, China.

4 Beijing National Laboratory for Condensed Matter Physics, Institute of Physics, Chinese Academy of Sciences, Beijing, China.

5 Key Laboratory of Structural Malformations in Children of Zhejiang Province, Wenzhou, China.

6 Pediatric Research Institute, Pediatric Interdisciplinary Consortium, The Second Affiliated Hospital and Yuying Children's Hospital of Wenzhou Medical University, Wenzhou, China.

**Corresponding author:** Changmin Shao (changmin_shao@163.com), Fangfu Ye (fye@iphy.ac.cn), Maoping Chu (chmping@hotmail.com)

#These authors contributed equally to this work.

**Figure S1.** Structure of the microfluidic device. A) Schematic illustration of the microfluidic device used for fiber fabrication. B) Photograph of the actual device (scale bar: 1 cm). C) Optical microscopy image showing the capillary outlet of the microfluidic device (scale bar: 200 μm).

**Figure S2.** Experimental setup for hydrogel fiber fabrication. A) Photograph showing the overall assembly of the device used for generating hydrogel hollow fibers (scale bar: 10 cm). B) Close-up view of the capillary outlet, which was shielded from light to prevent premature photocrosslinking (scale bar: 2 cm).

**Figure S3.** The effect of the internal phase flow rate (10-30 mL/h) on fiber dimensions (outer diameter and inner/outer diameter (ID/OD) ratio), with the external phase flow rate kept at 15 mL/h.

**Figure S4.** Rheological analysis showing the storage modulus (G′) and loss modulus (G′′) in different hydrogel groups as a function of frequency (0.1-10 Hz). Groups: PL (2.5% PEGDA), PM (5% PEGDA), and PH (7.5% PEGDA), all containing 10% GelMA.

**Figure S5.** Growth of HUVECs cultured for 48 h in different hydrogel groups. Groups: PL (2.5% PEGDA), PM (5% PEGDA), and PH (7.5% PEGDA), all containing 10% GelMA (scale bar: 100 μm).

**Figure S6.** Setup for fiber permeability characterization. A) Photograph of the experimental setup used for the permeability tests (scale bar: 10 cm). B) Close-up view showing the hollow fiber fixed within a PDMS mold for perfusion (scale bar: 5 cm).

**Figure S7.** Permeability tests using FITC-dextran. A) Representative fluorescence images showing the diffusion of 4 kDa FITC-dextran across acellular and cellular constructs at 0, 2, 4, 8, and 16 h. B) Representative fluorescence images showing the diffusion of 70 kDa FITC-dextran across acellular and cellular constructs at 0, 2, 4, 8, and 16 h. (scale bar: 500 μm)

**Figure S8.** Assessment of cell injury via LDH release. A) Comparison of LDH release levels between the Control and TNF-α groups. B) Comparison of LDH release levels between the HC and KD groups.

**Figure S9.** Evaluation of intracellular oxidative stress. A) Comparison of intracellular SOD activity between the Control and TNF-α groups. B) Comparison of intracellular SOD activity between the HC and KD groups.

**Figure S10.** Drug IC₅₀ curves. (A) IC₅₀ curve of atorvastatin. (B) IC₅₀ curve of MCC950.

**Figure S11.** Relative mRNA expression levels of indicated adhesion molecules and inflammatory cytokines (ICAM-1, VCAM-1, E-selectin, IL-1β, IL-6, IL-8, CX3CL1, CCL5, and MCP-1) in the HC, KD, and KD + ATOR groups.

**Figure S12.** Relative mRNA expression levels of indicated adhesion molecules and inflammatory cytokines (ICAM-1, VCAM-1, E-selectin, IL-1β, IL-6, IL-8, CX3CL1, CCL5, and MCP-1) in the HC, KD, and KD + MCC950 groups.

**Table S1.** Primer sequences for RT-qPCR analysis.

| Gene | Sequence (5‘ → 3’) | |
| --- | --- | --- |
| ICAM-1 | Forward sequence | ATGCCCAGACATCTGTGTCC |
|  | Reverse sequence | GGGGTCTCTATGCCCAACAA |
| VCAM-1 | Forward sequence | GGGAAGATGGTCGTGATCCTT |
|  | Reverse sequence | TCTGGGGTGGTCTCGATTTTA |
| E-selectin | Forward sequence | CGAGAGCTACACGTTCACGG |
|  | Reverse sequence | GGGTGTCGAGGGAAAAATAGG |
| IL-6 | Forward sequence | ACTCACCTCTTCAGAACGAATTG |
|  | Reverse sequence | CCATCTTTGGAAGGTTCAGGTTG |
| IL-8 | Forward sequence | TTTTGCCAAGGAGTGCTAAAGA |
|  | Reverse sequence | AACCCTCTGCACCCAGTTTTC |
| **IL-1β** | Forward sequence | ATGATGGCTTATTACAGTGGCAA |
|  | Reverse sequence | GTCGGAGATTCGTAGCTGGA |
| MCP-1 | Forward sequence | CAGCCAGATGCAATCAATGCC |
|  | Reverse sequence | TGGAATCCTGAACCCACTTCT |
| CX3CL1 | Forward sequence | ACCACGGTGTGACGAAATG |
|  | Reverse sequence | TGTTGATAGTGGATGAGCAAAGC |
| CCL5 | Forward sequence | CCAGCAGTCGTCTTTGTCAC |
|  | Reverse sequence | CTCTGGGTTGGCACACACTT |
| GAPDH | Forward sequence | GGAGCGAGATCCCTCCAAAAT |
|  | Reverse sequence | GGCTGTTGTCATACTTCTCATGG |

**Table S2.** Baseline demographic and clinical characteristics of the study population.

| Characteristics | | Healthy Controls  (n = 24) | Kawasaki Disease (n =29) | P value |
| --- | --- | --- | --- | --- |
| Age (months), mean ± SD | | 34.38±10.87 | 29.72±19.99 | 0.0659 |
| Sex (male / female) (n) | | (15/9) | (19/10) | >0.9999 |
| CAL status (Z score), n (%) | Normal (Z score < 2.0) | / | 27 (93.1%) | N/A |
|  | Dilation/Aneurysm (Z score ≥ 2.0) | / | 2 (6.9%) | N/A |

**Movie S1****(MP4 format).** Real-time observation of the hydrogel fiber photocrosslinking process.

**Movie S2 (MP4 format).** Three-dimensional (3D) reconstruction of the cell-laden fibers.

**Movie S3 (MP4 format).** Time-lapse video of the fiber permeability tests.
